# A Unified Framework for Classification and Segmentation of Ambiguous Dual-Type Lesions in Colonoscopic Images

**DOI:** 10.3390/bioengineering13060679

**Published:** 2026-06-11

**Authors:** Siqi Chen, Kun Jiang, Ruishi Lin, Xiufeng Su, Liyong Ma

**Affiliations:** 1 Department of Control Science and Engineering, Harbin Institute of Technology, Harbin 150001, China; 25b904075@stu.hit.edu.cn; 2 Suzhou Research Institute, Harbin Institute of Technology, Suzhou 215104, China; 3 Weihai Municipal Hospital, Cheeloo College of Medicine, Shandong University, Weihai 264299, China; jkdoctor@126.com (K.J.); suxiufeng1980@163.com (X.S.); 4 School of Information Science and Engineering, Harbin Institute of Technology, Weihai 264209, China; 23s030129@stu.hit.edu.cn

**Keywords:** colonoscopic images, joint classification and segmentation, dual-type lesions, semantic ambiguity, computer-aided diagnosis, deep learning

## Abstract

Accurate analysis of lesions in colonoscopic images is essential for computer-aided diagnosis. However, most existing methods are designed for single-lesion segmentation and assume a predefined lesion category, limiting their applicability in real-world scenarios where multiple lesion types exhibit similar visual characteristics. To address this issue, we propose a unified framework for the joint classification and segmentation of dual-type lesions in colonoscopic images, enabling simultaneous identification and localization of submucosal lesions and polyps/adenomas. The proposed method integrates joint supervision, context-aware feature enhancement, and ambiguity-aware optimization to improve consistency between semantic recognition and spatial delineation. In particular, a soft-label supervision strategy is introduced to alleviate semantic ambiguity, while an imbalance-aware loss design enhances segmentation accuracy and reduces false negative predictions. Extensive experiments on both private and public datasets demonstrate that the proposed method achieves superior performance compared with representative CNN- and transformer-based approaches. Notably, the method shows clear advantages in segmentation accuracy, localization precision, and robustness under challenging conditions. Ablation studies further confirm the effectiveness of each component in the proposed framework. These results indicate that the proposed approach provides an effective solution for dual-type lesion analysis and has the potential to assist clinical decision-making in gastrointestinal endoscopy.

## 1. Introduction

Medical image segmentation plays a fundamental role in computer-aided diagnosis, providing essential support for clinical decision-making, treatment planning, and disease monitoring [1]. In gastrointestinal endoscopy, accurate identification and delineation of lesions are particularly important for early detection and intervention. With the rapid development of deep learning, segmentation models have significantly outperformed traditional rule-based approaches in terms of robustness and accuracy [2].

Most existing studies in colonoscopic image analysis focus on single-lesion segmentation, particularly polyp detection, where the lesion category is assumed to be known in advance [3]. Early methods for colonic polyp segmentation mainly relied on rule-based image processing strategies, including thresholding [4], edge detection [5], region growing [6], and morphological operations [7]. Although these approaches are relatively simple to implement and computationally efficient, they largely depend on hand-crafted feature extraction, making it difficult to maintain stable segmentation performance in complex colonoscopic images.

With the advancement of deep learning, research attention has gradually shifted toward supervised segmentation frameworks based on encoder–decoder architectures. By learning in an end-to-end manner, these methods progressively integrate high-level semantic information with low-level spatial details, thereby significantly improving pixel-wise delineation of polyp regions. Representative works, such as U-Net and its subsequent variants, have established the fundamental technical paradigm for this research direction [8], including U-Net++ [9], the DeepLab series [10], and other related extensions [11]. Building upon this paradigm, recent studies have further explored multi-scale representation, boundary recovery, and cross-level feature fusion. For example, PraNet places greater emphasis on recovering ambiguous lesion boundaries [12], Polyp-PVT enhances global semantic modeling through Transformer-based representations [13], and XCC-Net improves segmentation performance through structural optimization [14]. Although these methods have achieved strong performance on public benchmarks, they remain limited in more realistic clinical scenarios, where different lesion types may coexist and exhibit similar visual appearances. In gastrointestinal endoscopy, some precancerous abnormalities, dysplasia, and cancerous lesions can appear visually similar, which increases the difficulty of reliable lesion analysis [15].

The key difficulty in this setting lies in the semantic ambiguity between lesion types and the coupling between classification and segmentation objectives [16]. When lesion categories are unknown and visually similar, models optimized solely for pixel-wise segmentation tend to focus on region coverage while neglecting category discrimination, leading to inconsistent predictions between lesion identity and spatial localization [17]. Therefore, classification and segmentation should not be treated as independent tasks, but rather jointly modeled to exploit their intrinsic complementarity, since shared representations can exploit the complementary information between the two tasks and improve both analytical capability and prediction consistency. This rationale is supported by recent studies on multi-task medical image analysis, which show that joint learning of related tasks can leverage their intrinsic correlations to improve performance and generalization [18].

Recent studies have explored joint learning frameworks that integrate classification and segmentation into a unified model. Tang et al. [19] proposed TransMT-Net, which unifies lesion classification and region segmentation within a single framework for gastrointestinal endoscopic image analysis. Wu et al. [20] developed ELNet to jointly perform lesion classification and region segmentation for esophageal lesion analysis. Zhou et al. [21] constructed a multi-task framework for automatic breast ultrasound images, further demonstrating the synergistic value between classification and segmentation. Barzegar and Khan [2] further designed SemiSeg-CAW for ultrasound image analysis, where a classification branch was introduced to facilitate multi-task collaborative optimization. These approaches demonstrate that global semantic information from classification can provide useful constraints for spatial prediction, while segmentation can enhance fine-grained localization. However, in colonoscopic applications, existing studies still mainly focus on polyp-related tasks [22], there remains limited research specifically targeting ambiguous dual-type lesion analysis in colonoscopic images, where both category discrimination and boundary delineation are critical.

To address these challenges, this paper proposes a jointly supervised framework for dual-type lesion analysis in colonoscopic images, which unifies classification and segmentation within a single learning paradigm. The proposed method is designed from three complementary perspectives, including input representation, network architecture, and optimization strategy, to improve the consistency between lesion recognition and spatial localization.

The main contributions of this work can be summarized as follows:

(1) Task-level contribution: We formulate a unified classification-segmentation framework for dual-type colonoscopic lesions, enabling the model to handle scenarios where lesion categories are unknown and visually ambiguous, rather than assuming a single predefined lesion type.
(2) Representation and architecture design: We introduce a jointly supervised encoder–decoder framework with auxiliary supervision and lightweight context enhancement, which improves the utilization of shallow features and enhances boundary-aware representation for accurate lesion delineation.
(3) Optimization strategy: We develop a joint optimization objective that integrates smoothed cross-entropy, Gaussian soft labels, Tversky loss, and false negative surrogate loss, allowing the model to simultaneously improve category discrimination, segmentation quality, and robustness against under-segmentation.

Extensive experiments on both private and public datasets demonstrate that the proposed method achieves superior performance in terms of lesion recognition, segmentation accuracy, and localization consistency, highlighting its potential for assisting clinical decision-making in gastrointestinal endoscopy.

The remainder of this paper is organized as follows. Section 2 reviews the related work. Section 3 presents the proposed method. Section 4 describes the experimental settings and results. Section 5 provides a discussion of the findings. Finally, Section 6 concludes the paper.

## 2. Related Work

For the classification and segmentation of dual-type lesions in colonoscopic images, related studies can be reviewed from two main perspectives. On the one hand, most existing endoscopic lesion segmentation methods are developed under single-lesion scenarios, mainly focusing on lesion region extraction, boundary recovery, and multi-scale feature modeling. On the other hand, recent studies have begun to explore joint modeling of classification and segmentation, aiming to integrate global semantic information and local spatial information within a unified learning framework.

### 2.1. Single-Lesion Endoscopic Lesion Segmentation

Automatic lesion segmentation in colonoscopic images has been extensively studied, particularly for polyp detection and delineation. Existing methods can generally be categorized into three directions [23].

The first group of methods is based on encoder–decoder architectures, which perform pixel-wise lesion region recovery by progressively integrating high-level semantic information with low-level spatial details. This type of method constitutes the basic technical framework of current endoscopic lesion segmentation. PolypSegNet modifies the network connections and feature transmission strategy of the conventional encoder–decoder architecture to improve fine-grained segmentation of polyp regions [24]. PSNet further introduces a dual-encoder and dual-decoder structure to enhance the complementary utilization of hierarchical features, thereby improving pixel-wise representation of complex polyp regions [25]. The second group of methods introduces attention mechanisms, context enhancement modules, or lightweight backbones into the basic segmentation framework to improve the modeling of complex background interference and locally discriminative features. Zhang et al. [26] enhanced contextual perception across regions of different scales through an adaptive context selection mechanism, allowing the model to better handle polyps with large size variations. Wei et al. [27] employed a shallow attention mechanism to suppress background noise and strengthen the representation of high-resolution shallow features for small polyps and weak-boundary regions. The third group of methods pays greater attention to multi-scale representation, boundary recovery, and cross-level feature interaction, aiming to alleviate the segmentation difficulties caused by scale variation, irregular morphology, and ambiguous boundaries of polyps. Liu et al. [28] strengthened the interaction among features at different levels through multi-level feature fusion and attention mechanisms, thereby enhancing the collaborative representation of polyp regions and detailed structures. EENet explicitly incorporates an edge enhancement mechanism into the segmentation network and improves the quality of polyp contour prediction through boundary-sensitive features [29]. Related reviews have shown that mainstream polyp segmentation methods have continuously evolved along these directions and achieved steady progress on public datasets [30].

Although existing methods have established relatively mature technical routes for single-lesion region segmentation, most of them assume that the lesion category to be segmented is known before modeling. Therefore, their performance improvements mainly lie in lesion region coverage, boundary recovery, and detailed delineation, while the explicit discrimination of lesion categories is less frequently considered. When the research target is extended from a single polyp to dual-type lesions with unknown categories and visually similar appearances, directly following this paradigm may be insufficient to support the joint modeling requirements of category discrimination and region segmentation.

### 2.2. Methods for Dual-Lesion Classification and Region Segmentation

To overcome the limitations of single-task learning, recent studies have explored joint frameworks that integrate classification and segmentation within a unified model. These approaches aim to leverage global semantic information from classification to guide spatial localization, while using segmentation to refine fine-grained feature representations. Various strategies have been proposed, including shared encoder architectures, auxiliary classification branches, and attention-based feature fusion mechanisms.

Zhu et al. [31] proposed the DSI-Net for joint classification and segmentation of wireless capsule endoscopy images. Different from simple parameter sharing, their method emphasized explicit cross-task interaction, showing that semantic recognition and lesion delineation can be mutually enhanced through task-specific feature exchange. Yu et al. [32] further developed a multi-task framework for esophageal lesion analysis, in which classification and segmentation were optimized within a unified architecture. Their study suggested that a shared feature space can simultaneously support lesion-level semantic understanding and region-level localization. Fan et al. [33] jointly addressed lesion localization and classification in breast ultrasound analysis, highlighting that category discrimination and spatial localization are intrinsically correlated rather than isolated objectives. Aumente-Maestro et al. [34] further constructed a unified segmentation classification framework for breast ultrasound tumor analysis, showing that multi-task collaboration can improve the overall consistency and reliability of lesion assessment.

Although these methods demonstrate the potential of multi-task learning, most of them are developed for general medical imaging tasks or scenarios with clear category distinctions. Limited attention has been paid to cases where lesion types are both visually similar and difficult to distinguish, such as in colonoscopic images involving submucosal lesions and polyps. In such ambiguous settings, existing joint learning frameworks may fail to achieve consistent optimization between classification and segmentation objectives. Therefore, a dedicated framework is required for ambiguous dual-type lesion analysis in colonoscopic images, enabling stable and accurate lesion category discrimination and region segmentation. Based on this motivation, the following section provides a detailed description of the proposed method from three aspects: preprocessing and soft supervision generation, the deeply supervised encoder–decoder architecture, and the joint optimization objective.

## 3. Method

### 3.1. Problem Formulation

In this study, we consider the task of joint lesion classification and segmentation in colonoscopic images, where the lesion category is not known a priori and may exhibit significant visual ambiguity.

Formally, let $D=\{(x_{i},{lc}_{i},m_{i}){\}}_{i=1}^{N}$ denote a dataset of colonoscopic images, where $x_{i}\in R^{H\times W\times 3}\;$ represents the input image, ${lc}_{i}\in \{1,\ldots ,C\}$ denotes the lesion category label, and $m_{i}\in \{0,\;1{\}}^{H\times W}$ is the corresponding pixel-wise segmentation mask. Here, C is the number of lesion categories.

The goal is to learn a mapping function, defined as follows:

(1) $$f_{\theta }:x\to \left(\hat{lc},\hat{m}\right),$$

parameterized by θ, which simultaneously predicts the lesion category $\hat{lc}$ and the segmentation mask $\hat{m}$.

Unlike conventional segmentation tasks that assume a single lesion type, the problem considered in this work is more challenging due to two key factors:

(1) Unknown lesion category

The model must infer the lesion type directly from the input image without prior knowledge, requiring strong global semantic understanding.

(2) Semantic ambiguity and visual similarity

Different lesion types may exhibit highly similar visual patterns in colonoscopic images, making it difficult to distinguish between them based solely on local features.

These challenges introduce a strong coupling between classification and segmentation tasks. Specifically, accurate segmentation relies on correct semantic understanding of the lesion type, while reliable classification depends on stable and consistent spatial-semantic feature representations. Therefore, optimizing these tasks independently may lead to suboptimal or inconsistent predictions.

To address this issue, we formulate the problem as a joint learning task, where classification and segmentation are optimized simultaneously under a unified framework. In the proposed implementation, this unified objective is instantiated through a jointly optimized deep-supervised architecture, where the overall training loss consists of a main decoding loss and an auxiliary supervision loss, and is defined as:

(2) $$L_{total}=L_{main}+\lambda_{aux}L_{aux},$$

where $L_{main}$ and $L_{aux}$ denote the losses of the main decoding branch and the auxiliary supervision branch, respectively, and $\lambda_{aux}$ is the balancing coefficient.

Under this joint learning setting, the key challenge is to design an effective mechanism that enables mutual enhancement between semantic recognition and spatial localization, while maintaining robustness to ambiguous lesion appearances. The proposed method addresses this challenge through coordinated design of input representation, network architecture, and optimization strategy, as detailed in the following sections.

### 3.2. Overview of the Proposed Framework

The overall framework is designed to address the joint classification–segmentation problem defined in Section 3.1, with a focus on improving consistency between semantic recognition and spatial localization under ambiguous lesion conditions.

**Figure 1:**
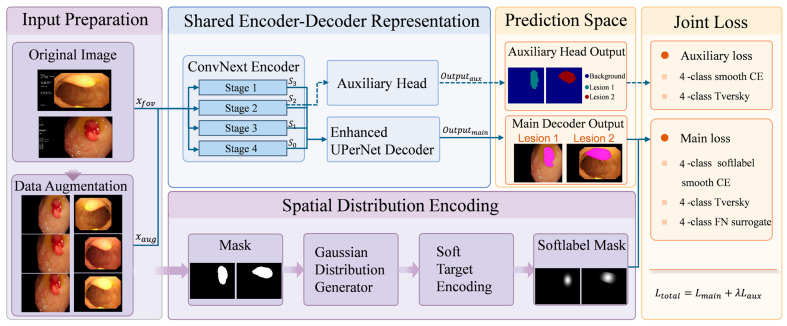
Overall architecture of the proposed model.

Given an input colonoscopic image x, the proposed model aims to simultaneously predict the lesion category $\hat{lc}$ and the corresponding segmentation mask $\hat{m}$. To achieve this, we adopt a unified encoder–decoder architecture with joint supervision, as illustrated in Figure 1.

Specifically, the framework consists of three main components: an input and supervision design stage for data augmentation and soft label generation, a network architecture stage for hierarchical representation learning and multi-scale feature aggregation, and an optimization strategy stage for joint optimization of classification and segmentation objectives.

In the first stage, the input image undergoes input preparation and spatial distribution encoding. Initially, effective field-of-view extraction is applied to suppress irrelevant background regions, followed by basic data augmentation to improve sample diversity and enhance robustness. Subsequently, a spatial distribution encoding process is introduced, where Gaussian-based soft labels are generated from the lesion masks to provide smooth and informative supervision signals. This design encourages the model to focus more consistently on lesion-relevant regions and establishes a foundation for subsequent optimization.

In the second stage, the processed image is fed into a shared encoder–decoder architecture for representation learning and prediction. First, the encoder extracts multi-level feature representations, capturing both local texture details and global contextual information, which are essential for distinguishing visually similar lesion types. Then, the extracted features are passed to the decoder, where multi-scale information is progressively fused to recover spatial resolution and refine lesion boundaries. To further enhance representation capability, a lightweight context enhancement module is incorporated to expand the receptive field and improve sensitivity to subtle structural variations. Meanwhile, auxiliary supervision is applied to intermediate feature maps to facilitate optimization and improve the utilization of shallow features. By enforcing supervision at multiple levels, the model is encouraged to learn more discriminative and robust representations.

In the final stage, an optimization strategy is introduced to jointly constrain classification and segmentation objectives. The overall training objective consists of a main loss and an auxiliary loss, corresponding to the outputs of the decoder and the auxiliary branch, respectively. The main loss simultaneously considers classification accuracy, segmentation quality, and false negative suppression, while the auxiliary loss provides additional guidance for intermediate representations. Furthermore, the soft labels generated in the first stage are incorporated into the classification-related supervision, enabling more stable and spatially consistent learning. Through this coordinated design, the optimization process effectively links input representation, feature learning, and supervisory signals, promoting mutual reinforcement between semantic discrimination and spatial delineation.

Overall, the proposed framework systematically integrates data preparation, representation learning, and joint optimization into a unified pipeline, where each stage is explicitly designed to support the collaborative modeling of semantic discrimination and spatial delineation. By aligning input supervision, feature learning, and optimization objectives toward a shared goal, the framework effectively addresses the challenges of semantic ambiguity and task coupling described in Section 3.1. In the following subsection, we first provide a detailed description of the Input and Supervision Design stage.

### 3.3. Input and Supervision Design

To improve model robustness and enhance learning under ambiguous lesion conditions, we design an input preprocessing and supervision strategy that integrates data normalization, augmentation, and soft-label guidance.

The first component is Input Preparation, which aims to reduce irrelevant background interference and improve the robustness of model training. All input colonoscopic images are first resized to a unified resolution to ensure a consistent input distribution. Before data augmentation, an effective field-of-view extraction step is applied, since colonoscopic images often contain dark borders, low-intensity peripheral regions, and other non-informative background content near the imaging boundary. To suppress such interference, only the valid imaging region is retained in both the original image and its corresponding mask.

Let the input colonoscopic image be denoted by x, The pixel value at the h-th row and w-th column of the input image is denoted as $x_{h,w}$. The image is first converted into a grayscale map G. Based on a grayscale threshold τ, a binary map B is generated to roughly separate the candidate tissue region:

(3) $$B\left(x_{h,w}\right)=\left\{\begin{array}{cc} 1, & G\left(x_{h,w}\right)>\tau ,\\ 0, & G\left(x_{h,w}\right)\leq \tau . \end{array}\right.$$

Then, an 8-connected component analysis is performed on B, and the set of foreground connected components is denoted as $\left\{{Fore}_{1},{Fore}_{2},\ldots ,{Fore}_{K}\right\}$. The largest connected component is selected as the final effective field-of-view region:

(4) $${Fore}^{*}=arg\;\underset{{Fore}_{k}}{max}|{Fore}_{k}|.$$

Accordingly, the binary mask of the effective field-of-view is defined as:

(5) $$M_{\mathrm{fov}}(x_{h,w})=\left\{\begin{array}{cc} 1, & x_{h,w}\in {Fore}^{*},\\ 0, & \mathrm{otherwise}. \end{array}\right.$$

The extracted FOV mask is then applied to both the original image and its annotation. For the image, the valid region is preserved as:

(6) $$x_{fov,h,w}=x_{h,w}\cdot M_{fov}\left(x_{h,w}\right),$$

where $x_{fov,h,w}$ denotes the colonoscopic image processed by the FOV mask $M_{fov}(x_{h,w})$, with all channels multiplied by the same mask. For the annotation mask $m_{h,w}$, it is first converted into a binary foreground mask:

(7) $$M_{bin}\left(m_{h,w}\right)=\left\{\begin{array}{cc} 1, & m_{h,w}>0,\\ 0, & m_{h,w}=0, \end{array}\right.$$

(8) $$m_{fov,h,w}=M_{bin}\left(m_{h,w}\right)\cdot M_{\mathrm{fov}}\left(x_{h,w}\right).$$

Through this preprocessing step, irrelevant border regions and non-informative background content can be effectively suppressed, allowing the model to focus more consistently on lesion-relevant regions.

After FOV-constrained preprocessing, basic data augmentation is further employed to improve sample diversity and enhance model generalization [35]. To improve robustness against variations in illumination, viewpoint, and lesion appearance, a series of lightweight augmentation techniques are applied during training, including random flipping, rotation, and intensity variation. These augmentations simulate realistic variability in endoscopic imaging and help prevent overfitting to specific visual patterns.

The second component is Spatial Distribution Encoding, which aims to construct spatially smooth and ambiguity-aware supervision signals for robust learning.

In conventional medical image segmentation tasks, ground-truth annotations are usually provided as binary masks. However, the actual lesion boundaries may contain annotation uncertainty, and hard boundaries may force the model to produce overly abrupt responses for pixels near lesion margins. This is not conducive to learning the continuous spatial structure between lesion interiors and boundary transition regions.

Since this study aims to jointly model lesion categories and pixel-level segmentation, an adaptive soft-label generation strategy based on a two-dimensional Gaussian distribution is further introduced. By constructing smoother spatial supervision signals, the soft labels encourage the model to learn the internal characteristics of different lesion regions and the structural patterns of their transition zones, which are often difficult to distinguish through direct visual inspection [36].

Let the FOV-constrained binary lesion mask be denoted as $m_{\mathrm{fov}}\in \{0,\;1{\}}^{H\times W}$, where $m_{\mathrm{fov}}(h,w)=1$ indicates lesion pixels and 0 indicates background. Considering that multiple disconnected lesion regions may exist in a single image, we first perform connected component analysis on $m_{\mathrm{fov}}$, obtaining a set of independent lesion instances $\left\{\Omega_{1},\Omega_{2},\ldots ,\Omega_{N}\right\}$. For the i-th lesion instance $\Omega_{i}$, its pixel coordinate set is defined as:

(9) $$\Omega_{i}=\left\{\left(h,w\right)|m_{fov}\left(h,w\right)=1,\;\left(h,w\right)\in \mathrm{the}\;i\text{-}\mathrm{th}\;\mathrm{connected}\;\mathrm{component}\right\}.$$

The geometric center $\mu_{i}=(\mu_{x,i},\mu_{y,i})$ of $\Omega_{i}$ is computed as:

(10) $$\left\{\begin{array}{c} \mu_{h,i}=\frac{1}{|\Omega_{i}|}\sum_{\left(h,w\right)\in \Omega_{i}}h,\\ \mu_{w,i}=\frac{1}{|\Omega_{i}|}\sum_{\left(h,w\right)\in \Omega_{i}}w. \end{array}\right.$$

Based on this center, a Gaussian distribution is constructed for each lesion instance. The spatial scale is adaptively determined by the maximum distance from pixels in $\Omega_{i}$ to the center:

(11) $$r_{i}=\underset{(h,w)\in \Omega_{i}}{max}\sqrt{\left(h-\mu_{h,i}{)}^{2}+(w-\mu_{w,i}{)}^{2}\right.}.$$

The standard deviation is defined as $\sigma_{i}=r_{i}/3$, and the corresponding Gaussian response is given by:

(12) $${Gauss}_{i}\left(h,w\right)=\exp\left(-\frac{\left(h-\mu_{h,i}{)}^{2}+(w-\mu_{w,i}{)}^{2}\right.}{2\sigma_{i}^{2}}\right).$$

To ensure that the soft label remains consistent with the original lesion region, the Gaussian response is constrained within the corresponding instance:

(13) $${\tilde{Gauss}}_{i}\left(h,w\right)=\left\{\begin{array}{cc} {Gauss}_{i}\left(h,w\right), & \left(h,w\right)\in \Omega_{i},\\ 0, & \mathrm{otherwise}. \end{array}\right.$$

The resulting soft label ${\tilde{Gauss}}_{i}(x,y)$ forms a continuous supervision distribution within each lesion region, where the response is highest at the center and gradually decays toward the boundary. Compared with conventional binary masks, this representation alleviates discontinuities at lesion boundaries and enables the model to learn smooth spatial transitions from lesion cores to edges.

Furthermore, by encoding spatial uncertainty and structural variation within lesion regions, the proposed soft-label mechanism provides more informative guidance for feature learning. This is particularly beneficial for ambiguous cases, where lesion categories share similar visual patterns and precise boundaries are difficult to define. Consequently, the model is encouraged to capture more robust intra-region representations rather than relying solely on sharp boundary cues, thereby improving consistency between semantic discrimination and spatial delineation. The following section presents the proposed network architecture designed to enhance semantic feature representation.

### 3.4. Deeply Supervised Encoder–Decoder Architecture

To effectively model the visual characteristics of colonoscopic images, we adopt an encoder–decoder architecture as the backbone of the proposed framework. The encoder is responsible for extracting hierarchical feature representations, while the decoder progressively fuses multi-scale features to generate high-resolution segmentation outputs.

Specifically, given the preprocessed input image $x_{\mathrm{fov}}$ and $x_{aug}$, the encoder produces a set of multi-level feature maps as:

(14) $$\{S_{0},S_{1},S_{2},S_{3}\}=E_{\theta_{e}}({cat(x}_{\mathrm{fov}},x_{aug})),$$

where $E_{\theta_{e}}$ denotes the encoder, and $S_{0}$, $S_{1}$, $S_{2}$, and $S_{3}$ represent feature maps from shallow to deep stages. This design provides strong representation capability for distinguishing visually similar lesion types, which is critical for accurate classification under semantic ambiguity [37].

**Figure 2:**
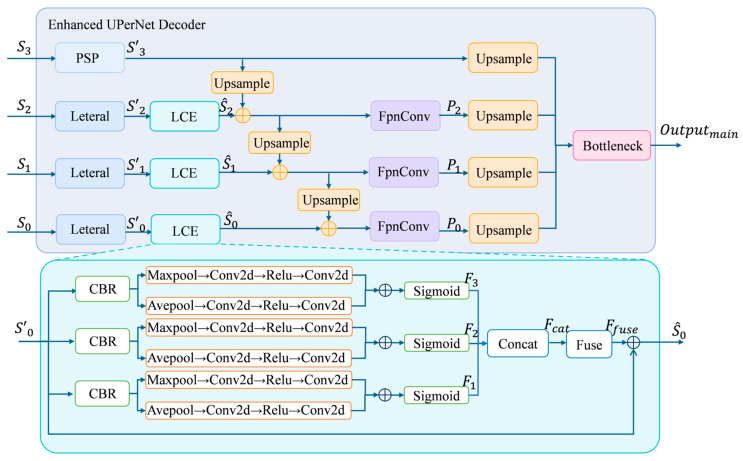
Integration of the Lightweight Context Enhancement module into lateral feature processing.

The decoder follows a multi-scale feature aggregation strategy, integrating information from different levels of the encoder to recover spatial resolution and refine lesion boundaries. In the initial decoder design, the deepest feature $S_{3}$ is first processed by a pyramid pooling module to capture global contextual information. Meanwhile, the lower-level features $S_{0}$, $S_{1}$, and $S_{2}$ are projected into a unified channel dimension through lateral transformations., and are then fused in a top-down manner to recover spatial resolution and refine lesion boundaries. However, in this design, only the highest-level feature $S_{3}$ benefits from explicit global context aggregation through the pyramid pooling module. The lateral features $S_{0}$, $S_{1}$, and $S_{2}$, which preserve richer spatial details, lack sufficient contextual modeling capability. This limitation becomes more critical in the considered task, where the model is required to jointly perform classification and segmentation under ambiguous dual-type lesion conditions. In such scenarios, relying solely on local information in shallow features may lead to insufficient discrimination between visually similar lesion types. To address this issue, we introduce a Lightweight Context Enhancement (LCE) module into the lateral feature processing stage, as illustrated in Figure 2. The key idea is to enrich the contextual representation of each feature level before top-down fusion, while preserving spatial details and maintaining computational efficiency.

Specifically, the LCE module is designed to capture multi-scale contextual information by combining convolutional operations with different receptive fields. By employing dilated convolutions with varying dilation rates, the module enables each feature map to aggregate information from multiple spatial ranges. This design allows the model to incorporate both local structural details and broader contextual cues, which are essential for distinguishing lesions with similar local appearances. Furthermore, instead of introducing heavy global attention or complex context modeling mechanisms, the LCE module adopts a lightweight multi-branch structure to balance representation capability and computational cost. This makes it suitable for integration into each lateral feature without significantly increasing model complexity.

The detailed operation of the LCE module is as follows. Given a lateral feature $S_{l}^{\prime }\in R^{C\times H\times W}$, three parallel convolutional branches are applied to extract features under different receptive fields as follows:

(15) $$F_{1}=\phi_{1}(S_{l}^{\prime }),$$

(16) $$F_{2}=\phi_{2}^{d=2}\left(S_{l}^{\prime }\right),$$

(17) $$F_{3}=\phi_{3}^{d=4}\left(S_{l}^{\prime }\right),$$

where $\phi_{1}(\cdot )$ denotes a 1×1 convolution, and $\phi_{2}^{d=2}(\cdot )$ and $\phi_{3}^{d=4}(\cdot )$ denote 3×3 dilated convolutions with dilation rates of 2 and 4, respectively. The outputs of the three branches are concatenated along the channel dimension as:

(18) $$F_{cat}=Concat\left(F_{1},F_{2},F_{3}\right),$$

and then fused through a 3×3 convolution as:

(19) $$F_{fuse}=\psi \left(F_{cat}\right),$$

where ψ(·) denotes the feature fusion and channel compression operation.

Finally, a residual connection is applied to obtain the enhanced feature as:

(20) $${\hat{S}}_{l}=F_{\mathrm{fuse}}+S_{l}^{\prime }.$$

This enhancement enriches the contextual representation of lateral features while preserving their spatial detail, enabling more effective integration of semantic and spatial information during feature fusion.

Given the inherent coupling between classification and segmentation tasks, as well as the imbalance of supervision across network depth, an auxiliary supervision branch is introduced on intermediate feature maps. In deep encoder–decoder architectures, high-level features typically receive more direct supervision from the final prediction, while shallow and intermediate features, despite containing richer edge, texture, and local structural information, are relatively under-supervised. This may limit the effectiveness of gradient propagation and hinder the learning of fine-grained spatial details.

To alleviate this issue, an auxiliary branch is attached to the intermediate feature $S_{2}$, where a lightweight FCN-based head is employed as the auxiliary prediction module. The FCN-based head adopts a simple convolutional structure to generate dense predictions from intermediate feature maps, enabling direct supervision at this level without introducing significant computational overhead.

During training, the auxiliary branch provides additional classification and segmentation supervision to intermediate representations. This design improves gradient propagation efficiency and encourages the network to better utilize local texture and boundary information, thereby enhancing the overall representation capability of the model.

Overall, the proposed architecture integrates hierarchical feature extraction, context enhancement, and auxiliary supervision into a unified framework. Hierarchical feature extraction enables multi-level representation of lesion characteristics, while context enhancement enriches spatial and structural information across different feature scales. The auxiliary supervision branch further improves gradient propagation and strengthens the learning of shallow features. Each component is designed to address the challenges identified in Section 3.1, including semantic ambiguity and task coupling, thereby improving the consistency between semantic discrimination and spatial delineation.

### 3.5. Optimization Strategy

The model is required to jointly perform classification and segmentation under ambiguous dual-type lesion conditions. In our implementation, this joint objective is realized through a combination of a main loss and an auxiliary loss. Both losses are defined on pixel-wise predictions and implicitly incorporate classification and segmentation signals. Specifically, classification is modeled as pixel-wise category prediction, while segmentation is enforced through region-level overlap and structural constraints.

To reflect their different roles in the network, the main and auxiliary losses are designed with different levels of strictness. The main loss is applied to the final prediction and aims to achieve accurate semantic discrimination, precise region delineation, and effective suppression of false negatives. In contrast, the auxiliary loss is imposed on intermediate features and adopts a simpler and more stable formulation to facilitate gradient propagation and improve shallow feature learning.

Based on this design, the overall loss function is defined as:

(21) $$L_{total}=L_{main}+\lambda_{aux}L_{aux},$$

where $L_{\mathrm{main}}$ and $L_{\mathrm{aux}}$ denote the losses of the main decoding branch and the auxiliary branch, respectively, and $\lambda_{\mathrm{aux}}$ is a balancing coefficient.

The main branch is responsible for generating the final prediction. Therefore, its objective is designed to jointly optimize classification accuracy, segmentation quality, and false negative suppression. The main loss is defined as:

(22) $$L_{main}=\lambda_{ce}L_{ce}+\lambda_{tv}L_{tv}+\lambda_{fn}L_{fn},$$

where $L_{ce}$ denotes the pixel-wise weighted smoothed cross-entropy loss, and $\lambda_{ce}$ is the corresponding weighting hyperparameter. $L_{tv}$ represents the Tversky loss, with $\lambda_{tv}$ controlling its contribution. $L_{fn}$ denotes the false negative surrogate loss, which is introduced to suppress missed lesion segmentation, and $\lambda_{fn}$ is its corresponding loss weight.

For classification-related supervision, a smoothed cross-entropy loss is adopted. Considering that manual annotations may contain uncertainty near lesion boundaries, label smoothing is introduced to improve robustness and generalization. In the main loss, the Gaussian-based soft labels proposed in Section 3.3 are further incorporated as pixel-wise weights, which emphasize informative regions within lesions and capture intra-lesion structures as well as transition patterns between regions. Let the logits output by the main branch be denoted as ${Pre}_{main}\in R^{B\times C\times H\times W}$, and the corresponding softmax probability be denoted as $p_{b,h,w,c}$. For each pixel location $\left(b,h,w\right)$, the ground-truth class label is denoted as ${\hat{l}}_{b,h,w}$. To alleviate the overly sharp supervision caused by hard labels, this study introduces a label smoothing strategy [38], and the smoothed target distribution is defined as:

(23) $$q_{b,h,w,c}=\left(1-\varepsilon \right)\mathrm{II}\left[c={\hat{lc}}_{b,h,w}\right]+\frac{\varepsilon }{C},$$

where ε denotes the label smoothing coefficient, and C represents the number of classes. Based on this formulation, the smoothed cross-entropy loss for a single pixel is computed as:

(24) $$l_{ce}\left(b,h,w\right)=-\sum_{c=1}^{C}q_{b,h,w,c}\log p_{b,h,w,c}.$$

Furthermore, the Gaussian soft label defined in Equation (13) is used to construct the pixel-level weight map ${\tilde{Gauss}}_{b,h,w}$, which is introduced to weight the smoothed cross-entropy loss. Accordingly, the weighted smoothed cross-entropy term in the main branch is written as:

(25) $$L_{ce}=\frac{1}{BHW}\sum_{b=1}^{B}\sum_{h=1}^{H}\sum_{w=1}^{W}{\tilde{Gauss}}_{\left(b,h,w\right)}l_{ce}\left(b,h,w\right).$$

For segmentation supervision, the Tversky loss is employed to explicitly balance false-positive and false-negative errors, which allows flexible control over these two types of errors [39], which is particularly important in medical segmentation tasks where under-segmentation is often more critical than over-segmentation. The logits output by the main branch are denoted as ${Pre}_{main}\in R^{B\times C\times H\times W}$. A softmax operation is first applied to obtain the predicted probability that each pixel belongs to class c:

(26) $$p_{b,c,i}=\frac{\exp\left({Pre}_{{main}_{b,c,i}}\right)}{\sum_{k=1}^{C}exp\left({Pre}_{{main}_{b,k,i}}\right)},$$

where b denotes the sample index in the batch, c denotes the class index, and i denotes the flattened pixel index. The corresponding ground-truth label is represented in one-hot form as ${\hat{m}}_{b,c,i}\in \{0,\;1\}$. For each sample b and foreground class c, the true positive, false positive, and false-negative are defined as:

(27) $$TP_{b,c}=\sum_{i}p_{b,c,i}{\hat{m}}_{b,c,i},$$

(28) $$FP_{b,c}=\sum_{i}p_{b,c,i}\left(1-{\hat{m}}_{b,c,i}\right),$$

(29) $$FN_{b,c}=\sum_{i}(1-p_{b,c,i}){\hat{m}}_{b,c,i},$$

Using the true positive, false positive, and false negative terms defined in Equations (27)–(29), the Tversky index for the c-th foreground class in the b-th sample is computed as:

(30) $$T_{b,c}=\frac{TP_{b,c}+\epsilon }{TP_{b,c}+\alpha FP_{b,c}+\beta FN_{b,c}+\epsilon },$$

where *ϵ* is a smoothing constant, and *α* and *β* control the penalty weights for false positives and false negatives, respectively.

Considering that not all foreground classes necessarily appear in the current batch, directly averaging over all classes may be affected by absent categories. Therefore, we average only over the foreground classes that are truly present in the current sample. The class presence mask is defined as:

(31) $${em}_{b,c}=\left\{\begin{array}{cc} 1, & \sum_{i}{\hat{m}}_{b,c,i}>0,\\ 0, & \mathrm{otherwise}. \end{array}\right.$$

Accordingly, the average Tversky index within the batch is formulated as:

(32) $$\bar{T}=\frac{\sum_{b}\sum_{c\in C_{fg}}{em}_{b,c}T_{b,c}}{\sum_{b}\sum_{c\in C_{fg}}{em}_{b,c}+\epsilon },$$

where $C_{fg}$ denotes the set of foreground classes. Finally, the multi-class Tversky loss is defined as:

(33) $$L_{tv}=1-\bar{T}.$$

Although the Tversky loss focuses on region overlap, false negative errors are particularly critical in lesion analysis. Therefore, an FN surrogate loss is introduced to explicitly penalize under-segmentation by directly optimizing a soft recall measure:

(34) $$L_{fn}^{\left(b\right)}=1-\frac{\sum_{h,w}p_{b,g_{b},h,w}\mathrm{II}\left[{\hat{lc}}_{b,h,w}={lc}_{b}\right]}{\sum_{h,w}\mathrm{II}[{\hat{lc}}_{b,h,w}={lc}_{b}]+\epsilon },$$

where $lc_{b}$ denotes the ground-truth lesion category of the current image. The overall false negative surrogate loss is then obtained by averaging the sample-wise FN surrogate loss in Equation (34) over all samples containing foreground lesions:

(35) $$L_{fn}=\frac{1}{B_{fg}}\sum_{b\in B_{fg}}L_{fn}^{\left(b\right)},$$

where $B_{fg}$ denotes the set of samples containing foreground lesions in the current batch. This term is essentially equivalent to directly optimizing the soft recall over the ground-truth lesion regions. Therefore, it can more specifically suppress false negative regions and improve the complete recovery of lesion areas.

The auxiliary branch provides direct supervision for intermediate features rather than performing final prediction. Since shallow features primarily encode low-level structures and are updated more slowly during training, a simpler and more stable objective is adopted as:

(36) $$L_{aux}=\lambda_{auxce}L_{auxce}+L_{tv},$$

where $\lambda_{auxce}$ denotes the weight of the smoothed cross-entropy loss in the auxiliary head, and $L_{auxce}$ represents the smoothed cross-entropy loss used for auxiliary supervision. The auxiliary smoothed cross-entropy loss is calculated as:

(37) $$L_{ce}=\frac{1}{BHW}\sum_{b=1}^{B}\sum_{h=1}^{H}\sum_{w=1}^{W}l_{ce}\left(b,h,w\right).$$

Compared with the main loss, Gaussian weighting is not applied in the auxiliary branch, as its purpose is to enhance feature separability and boundary awareness rather than enforce fine-grained spatial constraints. $L_{tv}$ denotes the Tversky loss, which is calculated in the same manner as that used in the main loss function.

Through the proposed joint optimization strategy, the model simultaneously enhances semantic discrimination, region-level consistency, and robustness against false negatives in the main branch, while improving intermediate feature learning via auxiliary supervision. The collaborative optimization of both branches not only improves final segmentation performance but also strengthens the representation of lesion textures, boundaries, and local structures. As a result, the framework achieves more stable and consistent predictions under ambiguous lesion conditions.

## 4. Experiment

### 4.1. Dataset

In this study, we focus on a dual-type lesion analysis task in colonoscopic images, where the model is required to simultaneously perform lesion category discrimination and pixel-wise segmentation. Unlike conventional single-lesion segmentation tasks, the lesion category is not assumed to be known a priori, and different lesion types may exhibit highly similar visual characteristics. This setting introduces additional challenges for both semantic recognition and spatial delineation. To comprehensively evaluate the proposed method, experiments are conducted on both a private dataset and a public dataset. These two datasets provide complementary characteristics in terms of data distribution and lesion variability.

The private dataset consists of colonoscopic images collected from clinical practice, focusing on dual-type lesion scenarios. Each image contains a single annotated lesion region, and all images are manually labeled with both lesion category and pixel-wise segmentation masks. The dataset includes two lesion categories, corresponding to submucosal lesions and polyp/adenoma-type lesions. Specifically, the dataset contains 2050 images of submucosal lesions and 2738 images of polyp/adenoma lesions, with a balanced distribution between the two categories. This dataset reflects real clinical conditions where lesion categories are not predefined and must be inferred from visual appearance.

To further validate the generalization ability of the proposed method, the Endoscopy Disease Detection and Segmentation 2020 dataset (EDD2020) [40] was adopted as the public external validation dataset. EDD2020 is a multi-center gastrointestinal endoscopy benchmark developed for disease detection and segmentation tasks. It contains 386 annotated endoscopic images collected from different anatomical sites, including the colon, esophagus, and stomach. The dataset covers five disease categories, namely Barrett’s esophagus (BE), suspicious lesions, high-grade dysplasia (HGD), cancer, and polyps. For each lesion instance, EDD2020 provides both bounding-box annotations for lesion localization and pixel-level instance segmentation masks for lesion delineation, making it suitable for evaluating both classification/localization and segmentation performance. Since the public release does not provide a predefined training/validation/test split, the dataset can be reorganized according to different experimental settings. Following the setting in Chavarrias-Solano et al. [41], the original categories are reorganized by merging cancer, high-grade dysplasia (HGD), and suspicious lesions into a single class, denoted as neoplasia. As a result, the task is reformulated as a dual-type lesion problem, consisting of polyp and neoplasia. After preprocessing, the dataset contains 122 polyp images and 210 neoplasia images, each annotated with both lesion category labels and segmentation masks. Compared with the private dataset, the public dataset exhibits greater diversity in imaging conditions, lesion appearance, and acquisition environments, providing a more challenging benchmark for evaluating model robustness.

The final data distribution is shown in Table 1. Despite differences in data sources, both datasets share a common challenge: lesion types are often visually similar and difficult to distinguish based solely on local appearance. Subtle differences in texture, structure, and contextual cues may determine the lesion category, while boundaries can be ambiguous due to imaging artifacts and annotation uncertainty.

These factors make it challenging to achieve consistent classification and segmentation, highlighting the importance of jointly modeling semantic discrimination and spatial delineation.

### 4.2. Implementation Details

All experiments were implemented using Python 3.8.20 and PyTorch 2.0.0, and were conducted on an NVIDIA RTX 5880 Ada GPU. The proposed model and comparison models were implemented based on the MMSegmentation framework. To ensure fair comparison and stable convergence, all models were initialized with publicly available ADE20K-pretrained weights provided by MMSegmentation, rather than being trained from scratch.

All input images and masks were resized to 512×512 before being fed into the network. The models were trained for 30 epochs with a batch size of 16. Adam was used as the optimizer. For the private dataset, the initial learning rate was set to $3\times 10^{-5}$, while for the public EDD2020 dataset, the initial learning rate was set to $1\times 10^{-3}$.

For the effective field-of-view extraction described in Section 3.3, the grayscale threshold τ was set to 30. For the joint optimization objective, the auxiliary loss weight was set to $\lambda_{\mathrm{aux}}=0.2$. In the main loss, the weights of the smoothed cross-entropy loss, Tversky loss, and FN surrogate loss were set to $\lambda_{\mathrm{ce}}=0.67$, $\lambda_{\mathrm{tv}}=1.0$, and $\lambda_{\mathrm{fn}}=0.4$, respectively. The label smoothing coefficient was set to ε=0.05. For the Tversky loss, the smoothing constant was set to $\epsilon =10^{-6}$, and the false-positive and false negative weighting parameters were set to α=0.3 and β=0.7, respectively. In the auxiliary loss, the weight of the auxiliary smoothed cross-entropy term was set to $\lambda_{\mathrm{auxce}}=0.67$.

### 4.3. Comparison with Representative Segmentation Methods

To evaluate the effectiveness of the proposed method, we compare it with several representative semantic segmentation models, including FCN, PSPNet, DeepLabv3+, UPerNet, SegFormer, Swin Transformer, and SegNeXt. These methods cover different technical paradigms, including classical convolutional segmentation networks, context aggregation frameworks, Transformer-based segmentation models, and mask prediction-based architectures. Therefore, they provide a comprehensive comparison for evaluating the proposed method under the dual-type lesion analysis setting.

FCN [42] is one of the earliest fully convolutional frameworks for semantic segmentation. It replaces fully connected layers with convolutional operations and performs pixel-wise prediction through upsampling and skip connections, establishing the foundation for subsequent segmentation networks.

PSPNet [43] introduces a pyramid pooling module to aggregate contextual information at multiple scales. By incorporating global prior information into pixel-wise prediction, PSPNet improves scene parsing and region-level understanding in complex images.

DeepLabv3+ [10] is a representative encoder–decoder segmentation framework. It employs atrous convolution and atrous spatial pyramid pooling to capture multi-scale contextual information, while the decoder helps recover spatial details and refine object boundaries.

UPerNet [44] combines pyramid pooling with a feature pyramid structure. It integrates global context from high-level features and progressively fuses multi-level features in a top-down manner, making it a representative framework for multi-scale semantic segmentation.

SegFormer [45] is an efficient Transformer-based segmentation model. Its hierarchical Transformer encoder extracts multi-scale features, and its lightweight MLP decoder performs feature fusion with relatively low computational complexity.

Swin [46] constructs hierarchical visual representations through shifted-window self-attention. By enabling cross-window information interaction while controlling computational cost, it serves as a strong backbone for dense prediction tasks.

SegNeXt [47] is a convolutional segmentation framework designed to improve contextual modeling through convolutional attention and large-kernel representation. It aims to achieve strong segmentation performance with efficient computation.

Since the proposed task involves both lesion-category discrimination and pixel-wise lesion delineation, four evaluation metrics are adopted to comprehensively assess model performance: lesion recognition accuracy, segmentation error, center localization error, and abnormal region localization Dice.

For lesion-category discrimination, we use the class-wise lesion recognition accuracy. For class c, it is defined as:

(38) $${Acc}_{c}=\frac{\sum_{n=1}^{N}\mathrm{II}\left({lc}_{n}=c,{\hat{lc}}_{n}=c\right)}{\sum_{n=1}^{N}\mathrm{II}\left({lc}_{n}=c\right)},$$

where ${lc}_{n}$ and ${\hat{lc}}_{n}$ denote the ground-truth and predicted lesion categories of the n-th image, respectively. A higher ${\mathrm{Acc}}_{c}$ indicates better lesion recognition performance.

For segmentation evaluation, we use segmentation error to measure the degree of missed lesion regions. For class c, the segmentation error is defined as:

(39) $${Segerr}_{c}=\frac{1}{N_{c}}\sum_{n_{c}=1}^{N_{c}}(\frac{1}{{Num}_{n_{c}}}\sum_{num=1}^{{Num}_{n_{c}}}\frac{{FN}_{num}^{n_{c}}}{{GT}_{num}^{n_{c}}+\varepsilon }),$$

where ${Num}_{n_{c}}$ denotes the number of lesions in the $n_{c}$-th image of class c, ${FN}_{num}^{n_{c}}$ denotes the missed segmentation area of the num-th lesion in the $n_{c}$-th image of class c, and ${GT}_{num}^{n_{c}}$ denotes the actual pixel area of the num-th lesion in the same image.

To evaluate localization accuracy, we calculate the center localization error. For class c, it is defined as:

(40) $${Cenerr}_{c}=\frac{1}{N_{c}}\sum_{n_{c}=1}^{N_{c}}(\frac{1}{{Num}_{n_{c}}}\sum_{num=1}^{{Num}_{n_{c}}}\frac{\sqrt{{\left(x_{p,num}^{\;n_{c}}\;-\;x_{g,num}^{\;n_{c}}\right)}^{2}+{\left(y_{p,num}^{\;n_{c}}\;-{\;y}_{g,num}^{\;n_{c}}\right)}^{2}}}{Diag^{\;n_{c}}+\varepsilon }),$$

where $\left(x_{p,num}^{\;n_{c}},y_{p,num}^{\;n_{c}}\right)$ denotes the centroid coordinates of the best-matched predicted instance, and $\left(x_{g,num}^{\;n_{c}},y_{g,num}^{\;n_{c}}\right)$ denotes the centroid coordinates of the corresponding ground-truth lesion instance. ${Diag}^{\left(n_{c}\right)}$ represents the diagonal length of the $n_{c}$-th image, which is used to normalize the center deviation.

Finally, abnormal region localization accuracy is evaluated using localization Dice. For class c, it is calculated as:

(41) $${LocDice}_{c}=\frac{1}{N_{c}}\sum_{n_{c}=1}^{N_{c}}(\frac{1}{{Num}_{n_{c}}}\sum_{num=1}^{{Num}_{n_{c}}}\frac{{2IoU}_{num}^{n_{c}}}{1+{IoU}_{num}^{n_{c}}}),$$

where ${IoU}_{num}^{\left(n_{c}\right)}$ denotes the intersection over union between the num-th ground-truth lesion instance in the $n_{c}$-th image and its best-matched predicted instance.

In addition to class-wise results, the average performance across lesion categories is reported for all four metrics. For Acc and LocDice, higher values indicate better performance, whereas lower values are preferred for SegErr and CenErr.

Based on the above comparison methods and evaluation metrics, quantitative experiments were conducted on both the private dataset and the public EDD2020 dataset. Table 2 reports the class-wise and average performance on the private dataset. In the table, ↑ indicates that a higher value represents better performance, whereas ↓ indicates that a lower value represents better performance.

As shown in Table 2, the proposed method achieves strong and balanced performance on both lesion categories in the private dataset. For lesion recognition accuracy, most comparison models obtain relatively high accuracy for polyp/adenoma lesions, but their performance on submucosal lesions is generally lower. For example, SegFormer achieves the highest ACC of 0.9871 on polyp/adenoma lesions, and Swin reaches 0.9742, both showing strong recognition ability for this category. However, their ACC values on submucosal lesions decrease to 0.9007 and 0.9433, respectively. In contrast, the proposed method achieves 0.9797 on polyp/adenoma lesions and 0.9645 on submucosal lesions. It also shows better category balance than DeepLabv3+, whose ACC values are 0.9576 and 0.9362 for polyp/adenoma and submucosal lesions, respectively. This suggests that the proposed framework is more effective in maintaining stable lesion discrimination under visually ambiguous conditions.

In terms of segmentation error, the proposed method also shows clear advantages. Its SegErr values are 0.0955 for submucosal lesions and 0.0656 for polyp/adenoma lesions, both remaining below 0.10. By comparison, DeepLabv3+ achieves 0.2063 and 0.1132, while Swin achieves 0.1587 and 0.0783 on the two lesion categories, respectively. Although SegFormer achieves a relatively low SegErr of 0.0691 on polyp/adenoma lesions, its error on submucosal lesions increases to 0.1590. This indicates that existing methods tend to achieve good segmentation quality on one lesion type while sacrificing the other. In contrast, the proposed method maintains low segmentation error for both lesion categories, suggesting stronger robustness against missed lesion regions. This improvement can be attributed to the joint effect of Gaussian-based soft supervision, Tversky loss, and FN surrogate loss, which encourage more complete lesion recovery.

For center localization error, the proposed method obtains the lowest errors for both lesion categories, with CenErr values of 0.0368 for submucosal lesions and 0.0241 for polyp/adenoma lesions. By comparison, DeepLabv3+ achieves 0.0616 and 0.0416 on the two categories, while UPerNet reaches 0.0686 and 0.0332. Swin performs relatively better on polyp/adenoma lesions, with a CenErr of 0.0294, but its error on submucosal lesions increases to 0.0763. Similarly, SegFormer obtains 0.0273 on polyp/adenoma lesions but 0.0783 on submucosal lesions. These results indicate that although some models can achieve competitive localization on easier lesion types, they lack stability across both categories. In contrast, the proposed method achieves consistently low localization errors, demonstrating stronger spatial consistency. This benefit is likely related to the lightweight context enhancement module, which enriches contextual representation while preserving local structural details.

Similar observations can be found in abnormal region localization performance. The proposed method achieves LocDice values of 0.8802 for submucosal lesions and 0.9163 for polyp/adenoma lesions. These values are higher than those of DeepLabv3+, which obtains 0.8127 and 0.8820, and Swin, which obtains 0.8265 and 0.9011. Although SegFormer achieves a relatively competitive LocDice of 0.9067 on polyp/adenoma lesions, its performance on submucosal lesions remains much lower at 0.8134. PSPNet shows a similar imbalance, with 0.8835 on polyp/adenoma lesions but only 0.7803 on submucosal lesions. In contrast, the proposed method maintains high localization Dice values across both categories, indicating better regional consistency and stronger balance between lesion types.

**Figure 3:**
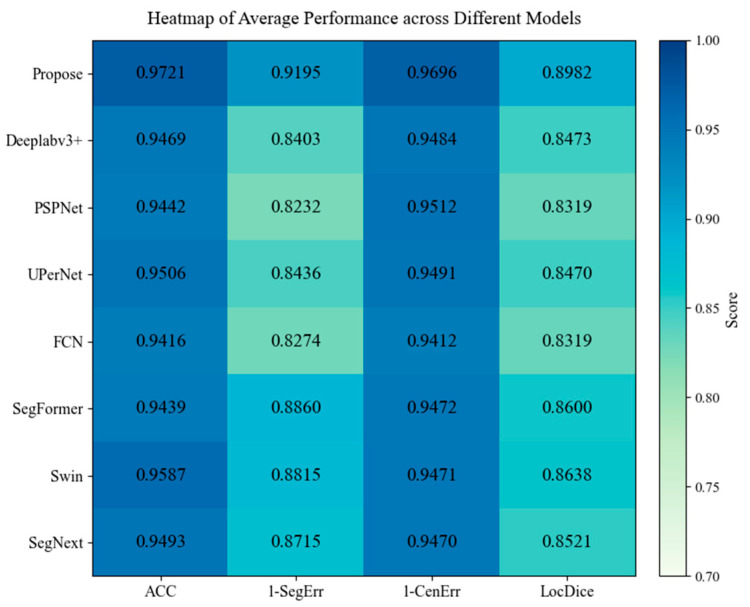
Heatmap of the average performance metrics on the private dataset.

While the class-wise results provide detailed comparisons for each lesion category, they do not directly reflect the overall performance differences among models. To provide a more intuitive and aggregated comparison, the average performance across the two lesion categories is further visualized as a heatmap, as shown in Figure 3. In the heatmap, the vertical axis represents different models, and the horizontal axis corresponds to the four evaluation metrics. Since higher values indicate better performance for ACC and LocDice, whereas lower values are preferred for SegErr and CenErr, the latter two metrics are transformed using 1−x to unify the interpretation direction, so that larger values consistently represent better performance.

As shown in Figure 3, the proposed method achieves the highest average ACC of 0.9721, indicating stronger and more stable lesion-category discrimination than all comparison models. In particular, it outperforms Swin, whose average ACC values is 0.9587. For segmentation completeness, the proposed method achieves the highest transformed 1−SegErr score of 0.9195, which is notably higher than those of SegFormer, and Swin, with scores of 0.8860, and 0.8815, respectively. Similar trends can be observed in center localization, where the proposed method obtains the highest transformed 1−CenErr score of 0.9696, outperforming PSPNet, DeepLabv3+, and UPerNet, whose scores are 0.9512, 0.9484, and 0.9491, respectively. For abnormal region localization, the proposed method also achieves the highest average LocDice of 0.8982, surpassing Swin and SegFormer, which obtain 0.8638 and 0.8600, respectively. Compared with these competitive baselines, the proposed framework shows stronger overlap consistency between predicted and ground-truth lesion regions, further demonstrating its robustness in challenging dual-type lesion scenarios.

**Figure 4:**
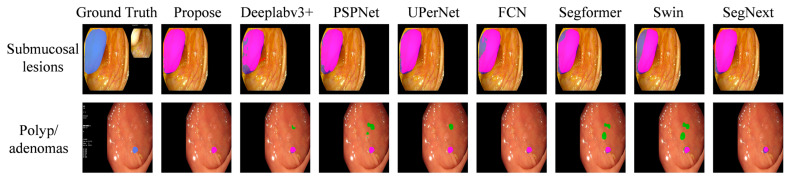
Qualitative comparison of segmentation results on the private dataset.

While the quantitative results and heatmap analysis provide objective comparisons across different evaluation metrics, they do not directly reflect the visual quality of lesion delineation. To further examine the segmentation behavior of different models, qualitative visualization results on the validation set are provided in Figure 4. These examples allow a more intuitive comparison of lesion coverage, boundary precision, and false-positive regions across different methods. In Figure 4, the visualization results of different models on the validation set are presented. For submucosal lesions, the predicted lesion regions are marked in red, the ground-truth regions are shown in blue, and their overlapping regions are shown in magenta. For polyp/adenoma lesions, the predicted lesion regions are marked in green, the ground-truth regions are shown in blue, and the overlapping regions are displayed as magenta.

From the visualization results, it can be observed that the proposed method achieves more complete lesion coverage for submucosal lesions, with fewer missed regions compared with the baseline methods. In particular, models such as SegFormer and Swin tend to miss relatively large lesion regions, especially in cases with ambiguous boundaries or irregular lesion structures. This observation is consistent with their higher SegErr values reported in Table 2. In contrast, the proposed method maintains more complete lesion delineation, indicating stronger robustness against under-segmentation.

For polyp/adenoma lesions, some baseline models, such as PSPNet and UPerNet, tend to produce over-segmentation, where surrounding normal tissue is incorrectly predicted as lesion regions. This leads to less precise boundary localization and increased false-positive regions. By comparison, the proposed method achieves more accurate lesion delineation and better boundary fitting, with fewer false-positive predictions and more consistent overlap with the ground-truth regions.

These qualitative observations are consistent with the quantitative and heatmap analyses, further confirming that the proposed framework achieves more balanced lesion recognition and more accurate lesion delineation under ambiguous dual-type lesion conditions. Overall, the private-dataset results support the effectiveness of the proposed framework. To further evaluate its generalization ability, experiments on the public EDD2020 dataset are conducted, and the results are presented in Table 3. In the table, ↑ indicates that a higher value represents better performance, whereas ↓ indicates that a lower value represents better performance.

Compared with the private dataset, this dataset is more challenging due to its smaller sample size, more heterogeneous imaging conditions, and the merged neoplasia category, which contains cancer, high-grade dysplasia, and suspicious lesions. Therefore, the classification boundary between polyp and neoplasia can be less stable, and the evaluation should consider not only classification accuracy but also segmentation completeness and localization quality.

In terms of classification accuracy, the proposed method does not achieve the highest average ACC. Its average ACC is 0.9230, which is lower than those of SegFormer, SegNeXt, UPerNet, and DeepLabv3+, with values of 0.9730, 0.9698, 0.9563, and 0.9532, respectively. However, high classification accuracy does not necessarily correspond to better segmentation quality. For example, SegFormer achieves a perfect ACC of 1.0000 on neoplasia, but its SegErr reaches 0.2041 for neoplasia and 0.3356 for polyp, indicating that accurate category prediction alone does not guarantee complete lesion delineation.

For segmentation error, the proposed method achieves the best average result among all models, with an average SegErr of 0.1481. Specifically, it obtains 0.1338 for neoplasia and 0.1624 for polyp. In comparison, SegNeXt obtains relatively high ACC values for both neoplasia and polyp, but its SegErr increases to 0.1707 and 0.2985, respectively. Compared with the proposed method, these values are approximately 27.58% higher for neoplasia and 83.81% higher for polyp. DeepLabv3+ also shows higher SegErr values of 0.2019 and 0.3050. These results suggest that the proposed method is more effective in reducing missed lesion regions, especially when lesion appearance is heterogeneous and category boundaries are ambiguous.

For center localization error, the proposed method achieves an average CenErr of 0.0740. This value is slightly higher than that of DeepLabv3+, which obtains 0.0698, but lower than most other comparison methods, including PSPNet, UPerNet, FCN, SegFormer, Swin, and SegNeXt, whose values are 0.1010, 0.0847, 0.0806, 0.1105, 0.2400, and 0.0800, respectively. On the polyp category, most models show relatively large center localization errors, while the proposed method maintains a CenErr of 0.1085. This result is close to DeepLabv3+, which obtains 0.0962, and lower than PSPNet, UPerNet, SegFormer, Swin, and SegNeXt, whose values are 0.1674, 0.1331, 0.1642, 0.1807, and 0.1231, respectively. Although DeepLabv3+ achieves slightly better center localization, its LocDice is lower than that of the proposed method, suggesting that accurate center prediction does not necessarily imply better lesion-region overlap.

For abnormal region localization, the proposed method achieves the highest average LocDice of 0.7380. It obtains 0.8036 for neoplasia and 0.6724 for polyp, outperforming DeepLabv3+ in overall average performance, whose corresponding values are 0.7601 and 0.6448. It also achieves a higher average LocDice than SegNeXt, which obtains 0.8083 for neoplasia and 0.6511 for polyp. Although PSPNet and UPerNet show slightly higher LocDice on neoplasia, their performance on polyp drops to 0.6194 and 0.6145, respectively. This indicates that the proposed method provides more balanced localization performance across the two lesion categories.

Overall, the public-dataset results show that the proposed method is not the strongest in classification accuracy, but it achieves better segmentation completeness and abnormal-region localization. This is important for the dual-type lesion analysis task, where the clinical utility of the model depends not only on predicting the lesion category but also on accurately delineating the lesion region. The improvement in SegErr and LocDice suggests that the proposed soft supervision, context-enhanced decoding, and false negative-aware optimization are beneficial for improving segmentation quality under heterogeneous and ambiguous public-dataset conditions.

**Figure 5:**
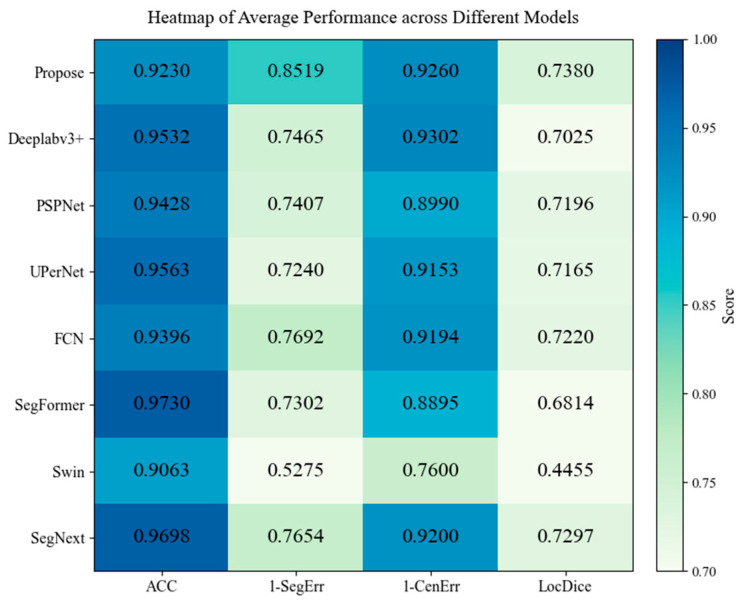
Heatmap of the average performance metrics on the public dataset.

To provide a more intuitive overview of the average performance on the public dataset, the four average metrics are further visualized using a heatmap, as shown in Figure 5. As in the private-dataset analysis, SegErr and CenErr are transformed into 1−SegErr and 1−CenErr, respectively, so that larger values consistently indicate better performance.

As shown in Figure 5, different models show distinct strengths across the four metrics. DeepLabv3+ obtains higher scores than the proposed method in ACC and 1−CenErr, with values of 0.9532 and 0.9302, respectively. However, the proposed method achieves clearly better scores in 1−SegErr and LocDice, reaching 0.8519 and 0.7380, compared with 0.7465 and 0.7025 for DeepLabv3+. This indicates that although DeepLabv3+ has a slight advantage in classification and center localization, the proposed method provides better lesion coverage and region-level localization.

To further compare the overall tendency across the four normalized metrics, we calculate a simple aggregate score by summing ACC, 1−SegErr, 1−CenErr, and LocDice. Under this auxiliary comparison, the proposed method obtains a total score of 3.4389, which is higher than the scores of DeepLabv3+ and SegNeXt, which are 3.3324 and 3.3849, respectively.

Therefore, the heatmap analysis further supports the observation from Table 3: the proposed method does not simply pursue higher classification accuracy, but achieves stronger overall lesion analysis performance by improving segmentation completeness and abnormal-region localization. This behavior is consistent with the design of the proposed framework, especially the use of spatial soft supervision and false negative-aware optimization.

**Figure 6:**
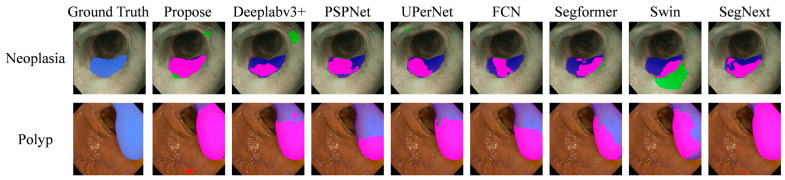
Qualitative comparison of segmentation results on the public dataset.

To further examine the segmentation behavior of different models beyond quantitative metrics, qualitative visualization results on representative validation samples from the public EDD2020 dataset are provided in Figure 6. Compared with numerical evaluation, these visual comparisons provide more intuitive evidence of lesion coverage, boundary delineation, and false negative regions, which are particularly important for evaluating model behavior under ambiguous lesion conditions. In Figure 6, the qualitative segmentation results of different models are presented for both neoplasia and polyp cases. For neoplasia lesions, the predicted regions are marked in green, the ground-truth regions are shown in blue, and the overlapping regions between prediction and ground truth are shown in magenta. For polyp lesions, the predicted regions are marked in red, while the ground-truth regions remain in blue, and the overlapping regions are shown in magenta.

For neoplasia cases, the proposed method demonstrates more complete lesion coverage, especially in peripheral and irregular regions of the lesion. Compared with the proposed method, several comparison models, such as PSPNet and UPerNet, tend to concentrate their predictions only on the central lesion area, while failing to recover the full lesion extent. Swin shows even larger deviations, with obvious under-segmentation and inaccurate regional responses. These observations are consistent with the quantitative SegErr results, where the proposed method achieves lower missed-region errors.

For polyp cases, the advantage of the proposed method becomes more evident. The predicted regions by the proposed framework show better consistency with the lesion boundaries and cover the lesion extent more completely. In comparison, SegFormer and PSPNet produce relatively conservative predictions, resulting in incomplete lesion coverage and missing peripheral structures. This phenomenon is particularly noticeable in irregular-shaped lesion regions, where accurate boundary recovery is more difficult.

It is worth noting that DeepLabv3+, which shows relatively competitive quantitative performance, does not exhibit the most visually extensive lesion coverage. Instead, its predictions are more concentrated in the central lesion area. This observation is consistent with its relatively high classification accuracy and competitive center localization performance, indicating that it can correctly identify lesion regions and localize lesion centers. However, its segmentation coverage remains less complete than that of the proposed method, which explains its inferior performance in SegErr and LocDice. This difference is clearly reflected in the visualization results.

Overall, the proposed method produces more complete lesion segmentation and more accurate boundary recovery, while the comparison methods tend to suffer from under-segmentation, especially in lesion corners, weak-boundary regions, and structurally complex areas. This behavior is likely related to the proposed soft-label supervision, which provides smoother spatial guidance near lesion boundaries, enabling the model to better capture intra-lesion structures and transition regions.

**Figure 7:**
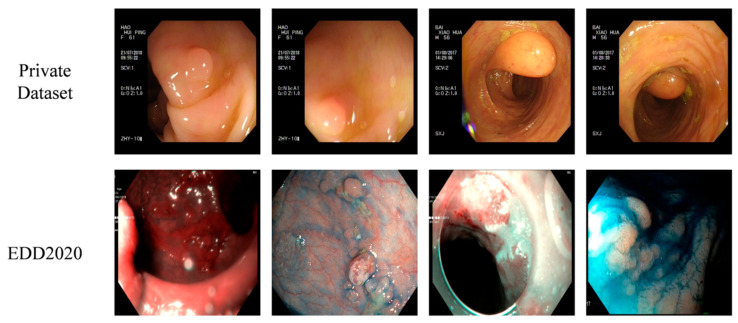
Examples of cross-dataset differences.

Although the proposed method achieves competitive performance on both datasets, the overall quantitative scores on EDD2020 are lower than those on the private dataset. This performance gap can be mainly attributed to several specific domain-shift factors rather than a single source of heterogeneity. To further illustrate the differences between the two datasets, representative examples from the private dataset and the public EDD2020 dataset are shown in Figure 7.

As shown in Figure 7, the private-dataset examples exhibit relatively consistent colonoscopic imaging characteristics, with more stable illumination, clearer lesion-background contrast, and less complex mucosal background interference. In contrast, the EDD2020 examples show greater variations in imaging conditions, including stronger illumination changes, more obvious specular reflection, more complex mucosal texture, and less distinct lesion boundaries. These differences are consistent with the fact that EDD2020 was collected from multiple institutions and multiple gastrointestinal anatomical sites, whereas the private dataset was obtained under relatively more consistent clinical acquisition conditions. Therefore, the model trained and evaluated on the private dataset can achieve higher scores, while direct evaluation on EDD2020 is more challenging due to acquisition-domain shift and anatomical-site shift.

Combining the results from both the private dataset and the public dataset, the proposed framework demonstrates consistently competitive performance against both CNN-based methods (such as FCN, PSPNet, DeepLabv3+, and UPerNet) and Transformer-based methods (such as SegFormer, Swin, and SegNeXt). Compared with CNN-based baselines, the proposed method achieves better lesion completeness and boundary consistency, while compared with Transformer-based baselines, it provides more stable performance across different lesion categories and avoids strong category imbalance.

These improvements can be mainly attributed to three aspects of the proposed framework. First, the joint learning strategy enables classification and segmentation to be optimized within a unified framework, improving consistency between semantic recognition and spatial delineation. Second, the soft-label supervision mechanism introduces smoother and more informative spatial guidance, which helps the model learn intra-lesion structures and boundary transitions more effectively. Third, the FN surrogate loss explicitly suppresses false negative regions, improving lesion completeness and reducing missed predictions.

More importantly, these advantages become more evident in challenging scenarios, including visually ambiguous lesions, small lesion regions, and unclear lesion boundaries, where conventional segmentation models often struggle to maintain stable performance. To further investigate the contribution of each component in the proposed framework, ablation experiments are conducted in the following section.

### 4.4. Ablation Study

To further validate the effectiveness of each component in the proposed framework, ablation experiments were conducted on the private dataset by progressively introducing the field-of-view (FOV) constraint, soft-label supervision, the enhanced decoder module, and auxiliary supervision. Starting from the baseline encoder–decoder framework, each component was incrementally added while keeping the other training settings unchanged. The quantitative results are summarized in Table 4. In the table, ↑ indicates that a higher value represents better performance, whereas ↓ indicates that a lower value represents better performance.

As shown in Table 4, the baseline model without additional modules achieves an average ACC of 0.9428, SegErr of 0.1682, CenErr of 0.0680, and LocDice of 0.8394. Although the baseline model provides a basic capacity for dual-type lesion classification and segmentation, its performance remains limited, especially for submucosal lesions. Specifically, the baseline model achieves an ACC of 0.9078, SegErr of 0.2152, CenErr of 0.0983, and LocDice of 0.7934 on submucosal lesions, indicating that this lesion type is more challenging due to its ambiguous appearance and less distinct boundary characteristics.

After introducing the FOV constraint, the average ACC increases from 0.9428 to 0.9637, while the average SegErr decreases from 0.1682 to 0.1267. Meanwhile, the average CenErr is reduced from 0.0680 to 0.0409, and the average LocDice improves from 0.8394 to 0.8726. The improvement is particularly evident for submucosal lesions, where ACC increases from 0.9078 to 0.9716, SegErr decreases from 0.2152 to 0.1444, CenErr decreases from 0.0983 to 0.0447, and LocDice increases from 0.7934 to 0.8606. These results indicate that the FOV constraint effectively suppresses irrelevant background interference and encourages the model to focus on lesion-relevant regions, thereby improving segmentation completeness and spatial localization. Although the ACC and LocDice of polyp lesions show slight fluctuations after introducing the FOV constraint, the overall performance is improved, suggesting that the FOV constraint mainly benefits the more ambiguous submucosal lesions.

By further incorporating soft-label supervision, the model achieves additional improvements in the overall evaluation metrics. The average ACC increases from 0.9637 to 0.9677, the average SegErr decreases from 0.1267 to 0.0842, and the average CenErr decreases from 0.0409 to 0.0314. The average LocDice also increases from 0.8726 to 0.8921. For polyp lesions, the improvement is substantial, with SegErr decreasing from 0.1089 to 0.0613 and LocDice increasing from 0.8845 to 0.9173. These results suggest that soft-label supervision can alleviate overly rigid decision boundaries and provide smoother spatial supervision, which is beneficial for improving segmentation robustness under ambiguous lesion appearances.

After adding the enhanced decoder module, the model further improves the average ACC from 0.9677 to 0.9684 and reduces the average SegErr from 0.0842 to 0.0805. For submucosal lesions, ACC increases from 0.9574 to 0.9645, SegErr decreases from 0.1071 to 0.0959, and LocDice increases from 0.8669 to 0.8707. These results indicate that the enhanced decoder strengthens contextual representation and improves the recovery of lesion structures, particularly for submucosal lesions. This suggests that the enhanced decoder improves segmentation error reduction and submucosal lesion representation.

Finally, after introducing auxiliary supervision, the model achieves the best overall performance in terms of ACC, CenErr, and LocDice. The average ACC increases from 0.9684 to 0.9721, the average CenErr decreases from 0.0358 to 0.0304, and the average LocDice increases from 0.8888 to 0.8982. The average SegErr remains at 0.0805, indicating that the segmentation error is maintained at the lowest level while localization consistency is further improved. For submucosal lesions, LocDice increases from 0.8707 to 0.8802, and CenErr decreases from 0.0425 to 0.0368. For polyp lesions, ACC increases from 0.9723 to 0.9797, CenErr decreases from 0.0291 to 0.0241, and LocDice increases from 0.9068 to 0.9163. These results demonstrate that auxiliary supervision provides effective optimization signals for intermediate features, improving gradient propagation and enhancing the learning of lesion-related structural details.

Overall, the ablation results demonstrate that each component contributes to the proposed framework from different aspects. The FOV constraint improves the model’s focus on valid endoscopic regions and substantially enhances the performance on submucosal lesions. Soft-label supervision improves segmentation robustness by providing smoother and more tolerant supervision under ambiguous lesion conditions. The enhanced decoder strengthens contextual representation and reduces segmentation error, especially for submucosal lesions. Auxiliary supervision further improves localization consistency and achieves the best overall performance. Compared with the baseline model, the final model improves the average ACC from 0.9428 to 0.9721, reduces SegErr from 0.1682 to 0.0805, reduces CenErr from 0.0680 to 0.0304, and improves LocDice from 0.8394 to 0.8982. These results validate the effectiveness and rationality of the proposed module design.

**Figure 8:**
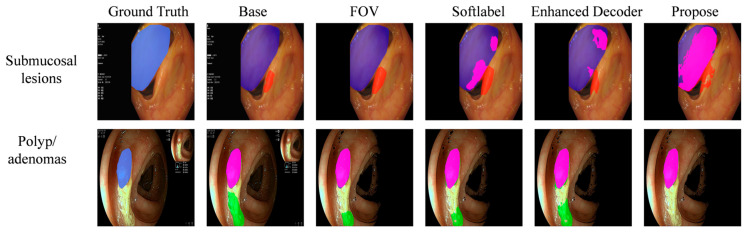
Representative qualitative results of the ablation study.

To further provide intuitive insight into how each module improves the segmentation performance, representative qualitative examples from the ablation study are shown in Figure 8. In Figure 8 the color-coded regions indicate the relationship between the ground truth and the predicted lesion areas. For submucosal lesions, the ground-truth regions are shown in blue, the overlapping regions between the prediction and the ground truth are shown in magenta, and the false-positive regions, namely areas predicted as lesions but not annotated as lesions in the ground truth, are shown in red. For polyp/adenoma lesions, the ground-truth regions are shown in blue, the overlapping regions between the prediction and the ground truth are shown in magenta, and the false-positive regions are shown in green.

As shown in the representative case of submucosal lesions, both the baseline model and the model with the FOV constraint fail to correctly detect the lesion region. After introducing soft-label supervision, the model begins to identify the lesion boundary, but a relatively large deviation is still observed, with part of the background being incorrectly segmented as a lesion. After the enhanced decoder is added, the predicted region becomes more concentrated around the lesion center, and the false-positive response in the background is reduced. With the further introduction of auxiliary supervision, the proposed model produces fewer misclassified regions, recovers a larger portion of the true lesion area, and yields a predicted segmentation center that is closer to the center of the ground truth. These observations suggest that soft-label supervision improves sensitivity to ambiguous lesion boundaries, while the enhanced decoder and auxiliary supervision further strengthen contextual discrimination and spatial localization.

For the polyp/adenoma case, the proposed model can effectively avoid the influence of illumination interference. In contrast, the other ablation settings show varying degrees of false-positive prediction in the reflective region, indicating that the specular highlight can be easily confused with lesion tissue. The final model successfully separates the true lesion region from the background, demonstrating that the proposed framework achieves better robustness to illumination variation and more reliable lesion-background discrimination.

## 5. Discussion

This study investigates joint classification and segmentation of dual-type lesions in colonoscopic images, with a particular focus on scenarios where lesion categories are visually ambiguous and difficult to distinguish. Unlike conventional approaches that assume a single lesion type, the proposed framework explicitly considers the coupling between semantic discrimination and spatial delineation, enabling more consistent lesion analysis in complex clinical settings.

### 5.1. Effectiveness of the Proposed Framework

The experimental results demonstrate that the proposed method achieves competitive and generally consistent improvements across multiple evaluation metrics, particularly in segmentation completeness and localization stability. These improvements can be attributed to the coordinated design of task formulation, feature representation, and optimization strategy.

First, formulating the problem as a unified classification–segmentation task allows the model to jointly learn lesion identity and spatial extent. This is especially important in ambiguous scenarios, where segmentation-only methods may focus on region coverage without sufficiently capturing category-related characteristics. By jointly considering semantic discrimination and spatial delineation, the model achieves better consistency between lesion category and lesion region.

Second, the integration of enhanced feature representation and auxiliary supervision improves the utilization of both high-level semantic features and low-level spatial details. The enhanced decoder module enriches contextual information during multi-scale fusion, while auxiliary supervision provides direct guidance to intermediate features. The ablation results show that these components contribute to reduced segmentation error and improved localization performance, especially for challenging lesion types.

Third, the proposed optimization strategy further improves robustness. Gaussian-based soft-label supervision provides spatially adaptive guidance under ambiguous lesion conditions, while the combination of Tversky loss and FN surrogate loss improves sensitivity to lesion regions and reduces missed segmentation. As a result, the model achieves a better balance between category discrimination, lesion-region recovery, and localization accuracy.

Overall, these components work in a complementary manner to address ambiguous lesion appearance and task coupling, leading to improved overall performance compared with representative segmentation methods.

### 5.2. Clinical Relevance and Practical Implications

From a clinical perspective, accurate identification and localization of different lesion types in colonoscopy are important for diagnosis, treatment planning, and follow-up management. In real-world scenarios, lesions such as submucosal lesions and polyps/adenomas may exhibit overlapping visual characteristics, making reliable differentiation challenging.

The proposed framework provides a potential solution by jointly predicting lesion category and spatial extent within a unified model. Such a joint analysis mechanism may assist clinicians in identifying lesion types while also assessing their precise locations and regions, thereby improving diagnostic efficiency and reducing the risk of missed or incomplete lesion interpretation.

Moreover, the improved segmentation robustness observed in both private and public datasets suggests that the method may be useful under challenging imaging conditions, such as ambiguous boundaries, low contrast, or atypical lesion appearance. These results indicate the potential of the proposed approach for computer-aided endoscopic diagnosis systems.

### 5.3. Limitations

Despite the promising results, several limitations should be noted. First, the classification performance, particularly on the public dataset, remains less stable than segmentation-related performance. Although the proposed method achieves strong results in segmentation error and localization Dice, its average classification accuracy does not exceed all comparison models. This indicates that the model’s semantic discrimination ability under cross-dataset or heterogeneous data conditions still requires improvement.

One possible reason is that the current framework mainly strengthens classification through supervision and optimization design, while the architectural components are more directly oriented toward segmentation and spatial representation. Therefore, category-discriminative feature learning still needs to be further enhanced in future work.

Second, the current study focuses on a dual-type lesion setting. Although this setting reflects clinically meaningful ambiguity between visually similar lesions, it does not fully cover the complexity of real-world colonoscopic scenarios involving multiple lesion categories and more diverse disease patterns. The scalability and generalization ability of the proposed framework still need to be validated on larger, multi-center, and multi-class datasets.

### 5.4. Future Work

Future work can be conducted in several directions. First, more effective category-discriminative representation learning should be explored to improve classification robustness, especially under cross-dataset and domain-shift conditions. This may involve introducing stronger semantic modeling mechanisms or domain adaptation strategies.

Second, the proposed framework can be extended from dual-type lesion analysis to multi-class gastrointestinal lesion analysis, enabling joint recognition and segmentation of a broader range of abnormalities. In addition, more comprehensive error case analysis and visualization studies should be conducted to better understand model behavior under different lesion types, boundary conditions, and image quality levels.

## 6. Conclusions

This paper addresses the challenging task of joint classification and segmentation of dual-type lesions in colonoscopic images, where lesion categories may be visually ambiguous and difficult to distinguish. Unlike conventional approaches that assume a single lesion type, the proposed framework jointly models semantic discrimination and spatial delineation within a unified learning paradigm.

To this end, a jointly supervised architecture is developed by integrating hierarchical feature representation, context enhancement, and ambiguity-aware optimization. By incorporating Gaussian-based soft-label supervision, auxiliary learning, and false negative-aware loss design, the proposed method improves the consistency between lesion identification and lesion-region delineation, particularly in challenging cases with unclear boundaries or overlapping visual characteristics.

Experiments on both private and public datasets demonstrate that the proposed method achieves strong performance in segmentation accuracy and localization stability, while maintaining competitive classification capability. The ablation results further confirm that the main components contribute complementary improvements to the overall framework.

Despite these promising results, classification performance under cross-dataset conditions remains a limitation, indicating the need for stronger semantic generalization. Future work will focus on enhancing category-discriminative representation learning, strengthening the interaction between classification and segmentation, and extending the framework to more complex multi-class clinical settings. The proposed framework has the potential to assist clinical decision-making in gastrointestinal endoscopy by providing more reliable lesion identification and region delineation.

## Figures and Tables

**Table 1:** Summary of the datasets used in this study.

Dataset	Type	Images
Private dataset	Polyp/Adenoma	2738
	Submucosal lesion	2050
EDD2020	Polyp	120
	Neoplasia	210

**Table 2:** Comparative experimental results on the private dataset.

	ACC↑	Segerr↓	Cenerr↓	LocDice↑	Models
Submucosal lesion	0.9645	0.0955	0.0368	0.8802	Propose
Polyp/Adenoma	0.9797	0.0656	0.0241	0.9163	
Average	0.9721	0.0805	0.0304	0.8982	
Submucosal lesion	0.9362	0.2063	0.0616	0.8127	Deeplabv3+
Polyp/Adenoma	0.9576	0.1132	0.0416	0.8820	
Average	0.9469	0.1597	0.0516	0.8473	
Submucosal lesion	0.9291	0.2443	0.0682	0.7803	PSPNet
Polyp/Adenoma	0.9594	0.1093	0.0293	0.8835	
Average	0.9442	0.1768	0.0488	0.8319	
Submucosal lesion	0.9362	0.2082	0.0686	0.8056	UPerNet
Polyp/Adenoma	0.9649	0.1045	0.0332	0.8884	
Average	0.9506	0.1564	0.0509	0.8470	
Submucosal lesion	0.9220	0.2338	0.0751	0.7841	FCN
Polyp/Adenoma	0.9613	0.1113	0.0425	0.8798	
Average	0.9416	0.1726	0.0588	0.8319	
Submucosal lesion	0.9007	0.1590	0.0783	0.8134	Segformer
Polyp/Adenoma	0.9871	0.0691	0.0273	0.9067	
Average	0.9439	0.1140	0.0528	0.8600	
Submucosal lesion	0.9433	0.1587	0.0763	0.8265	Swin
Polyp/Adenoma	0.9742	0.0783	0.0294	0.9011	
Average	0.9587	0.1185	0.0529	0.8638	
Submucosal lesion	0.9504	0.1619	0.066	0.8214	SegNext
Polyp/Adenoma	0.9483	0.0952	0.0400	0.8828	
Average	0.9493	0.1285	0.0530	0.8521	

**Table 3:** Comparative experimental results on the public dataset.

	ACC↑	Segerr↓	Cenerr↓	LocDice↑	Models
Neoplasia	0.9000	0.1338	0.0394	0.8036	Propose
Polyp	0.9459	0.1624	0.1085	0.6724	
Average	0.9230	0.1481	0.0740	0.7380	
Neoplasia	0.9333	0.2019	0.0435	0.7601	Deeplabv3+
Polyp	0.9730	0.3050	0.0962	0.6448	
Average	0.9532	0.2535	0.0698	0.7025	
Neoplasia	0.9667	0.1431	0.0345	0.8198	PSPNet
Polyp	0.9189	0.3754	0.1674	0.6194	
Average	0.9428	0.2593	0.1010	0.7196	
Neoplasia	0.9667	0.1671	0.0362	0.8185	UPerNet
Polyp	0.9459	0.3850	0.1331	0.6145	
Average	0.9563	0.2760	0.0847	0.7165	
Neoplasia	0.9333	0.1278	0.0403	0.8219	FCN
Polyp	0.9459	0.3337	0.1208	0.6221	
Average	0.9396	0.2308	0.0806	0.7220	
Neoplasia	1.0000	0.2041	0.0567	0.7536	Segformer
Polyp	0.9459	0.3356	0.1642	0.6093	
Average	0.9730	0.2698	0.1105	0.6814	
Neoplasia	0.8667	0.4905	0.2993	0.4469	Swin
Polyp	0.9459	0.4545	0.1807	0.4440	
Average	0.9063	0.4725	0.2400	0.4455	
Neoplasia	0.9667	0.1707	0.0368	0.8083	SegNext
Polyp	0.9730	0.2985	0.1231	0.6511	
Average	0.9698	0.2346	0.0800	0.7297	

**Table 4:** Ablation study results.

	ACC↑	Segerr↓	Cenerr↓	LocDice↑	Add
Submucosal lesion	0.9078	0.2152	0.0983	0.7934	Base
Polyp/Adenoma	0.9779	0.1211	0.0377	0.8854	
Average	0.9428	0.1682	0.0680	0.8394	
Submucosal lesion	0.9716	0.1444	0.0447	0.8606	FOV
Polyp/Adenoma	0.9557	0.1089	0.0372	0.8845	
Average	0.9637	0.1267	0.0409	0.8726	
Submucosal lesion	0.9574	0.1071	0.0394	0.8669	Softlabel
Polyp/Adenoma	0.9779	0.0613	0.0234	0.9173	
Average	0.9677	0.0842	0.0314	0.8921	
Submucosal lesion	0.9645	0.0959	0.0425	0.8707	Enhanced Decoder
Polyp/Adenoma	0.9723	0.0651	0.0291	0.9068	
Average	0.9684	0.0805	0.0358	0.8888	
Submucosal lesion	0.9645	0.0955	0.0368	0.8802	Auxiliary
Polyp/Adenoma	0.9797	0.0656	0.0241	0.9163	
Average	0.9721	0.0805	0.0304	0.8982	

## Data Availability

The private dataset used in this study is not publicly available due to privacy and ethical restrictions. The public dataset (EDD2020) is available at: https://ead2020.grand-challenge.org/ (accessed on 28 April 2026).
